# Anytime ECG Monitoring through the Use of a Low-Cost, User-Friendly, Wearable Device

**DOI:** 10.3390/s21186036

**Published:** 2021-09-09

**Authors:** Vincenzo Randazzo, Jacopo Ferretti, Eros Pasero

**Affiliations:** 1 Politecnico di Torino, 10129 Turin, Italy; jacopo.ferretti@polito.it (J.F.); eros.pasero@polito.it (E.P.); 2 Department of Surgical Sciences, Università degli Studi di Torino, 10124 Turin, Italy

**Keywords:** analog filters, atrial fibrillation, ECG WATCH, ECG, EKG, electrocardiogram, instrumentation amplifier, mobile healthcare, telemedicine

## Abstract

Every year cardiovascular diseases kill the highest number of people worldwide. Among these, pathologies characterized by sporadic symptoms, such as atrial fibrillation, are difficult to be detected as state-of-the-art solutions, e.g., 12-leads electrocardiogram (ECG) or Holter devices, often fail to tackle these kinds of pathologies. Many portable devices have already been proposed, both in literature and in the market. Unfortunately, they all miss relevant features: they are either not wearable or wireless and their usage over a long-term period is often unsuitable. In addition, the quality of recordings is another key factor to perform reliable diagnosis. The ECG WATCH is a device designed for targeting all these issues. It is inexpensive, wearable (size of a watch), and can be used without the need for any medical expertise about positioning or usage. It is non-invasive, it records single-lead ECG in just 10 s, anytime, anywhere, without the need to physically travel to hospitals or cardiologists. It can acquire any of the three peripheral leads; results can be shared with physicians by simply tapping a smartphone app. The ECG WATCH quality has been tested on 30 people and has successfully compared with an electrocardiograph and an ECG simulator, both certified. The app embeds an algorithm for automatically detecting atrial fibrillation, which has been successfully tested with an official ECG simulator on different severity of atrial fibrillation. In this sense, the ECG WATCH is a promising device for anytime cardiac health monitoring.

## 1. Introduction

The continuous pumping action of the heart is a fundamental need for human life; indeed, through contractions and relaxations, i.e., the cardiac cycle, it pumps the blood through the circulatory system vessels allowing the oxygenation of the body organs. This process is regulated by electrical impulses that stimulate different parts of the heart and should repeat constantly. Natural human aging may lead to irregularities in the heart pace causing cardiovascular diseases (CVDs), such as arrhythmias, myocardial ischemia or infarction. CVDs remain the most common cause of death worldwide. Around one third of all deaths are related to this group of pathologies, more than twice that caused by cancer, as well as more than all communicable, maternal, neonatal, and nutritional disorders combined [1,2,3,4]. According to statistics, the size of the elderly population is expected to grow substantially in the next few years; as a consequence, CVDs will follow the same trend [5]. In such a scenario, instrumentation and measurement represents a fundamental asset for cardiologists to understand patient conditions and perform diagnoses [6].

The standard procedure to analyze the heart’s state of health is the use of a multi-lead electrocardiograph, which records heart electrical activity through wet electrodes placed on the skin and visualizes it into a time graph, called an electrocardiogram (ECG) [7,8]. Generally, these machines perform high-resolution acquisitions and, for this reason, are quite expensive. ECG has been proven to be the most efficient way to diagnose CVDs [9,10,11]; indeed, the use of a multi-lead recording system provides, as output, a collection of signals, which represent different perspectives of the heart muscle’s electrical field allowing physicians to have a comprehensive view of the patient heart. In order to detect anomalies in the recorded ECG traces, both patients and doctors must be in the same room together with the electrocardiograph. Diseases characterized by sporadic events, such as the atrial fibrillation (A-fib), cannot be diagnosed if they do not occur exactly during ECG acquisition. Unfortunately, in a more realistic scenario, these pathologies may be latent for a very long time and, in the worst case, can kill people without any evident symptoms.

To overcome these limitations, different strategies have been proposed [12,13]. The most adopted solution is the Holter device [14,15,16,17] for the continuous monitoring (24 h–48 h) of CVD patients. In this way, cardiologists have at their disposal enough data to record even sporadic anomalies, and diagnosis could be performed in a timely manner. Unfortunately, they are non-wireless and expensive, limiting the number of patients they could be applied on; their recording autonomy is limited and, sometimes, not sufficient for discovering sporadic but very severe diseases. Finally, they cannot be used for real-time diagnosis because they need to record data and then, after the device removal from the patient’s body, ECG traces have to be inspected by a cardiologist.

In addition to devices already available in the market, which will be analyzed in detail in the next section, many proofs of concept have been proposed in literature for non-invasive, wearable, and reliable heart monitoring [18,19,20,21,22]. A diagnostic ECG device exploiting web-services to share recordings is introduced in [23], while [24] uses disposable electrodes and a built-in warning system. Sensing devices for acquiring multiple vital signs are presented in [25,26,27] and [28] for CVD remote monitoring and electromyography/electrocardiography, respectively. Conductive fabric is used in [29,30,31,32,33,34,35] to acquire ECG through sensors embedded in clothes, while armband and multi-ring are employed in [36,37,38], respectively. Smartphone-based devices are proposed in [39,40,41,42,43,44]. ECG recording devices with particular focus on low power are shown in [45,46,47,48]. In these two latter categories, it is found the ECG WATCH [49,50,51], which can record one-lead ECGs in just 10 s and, also, looks for silent atrial fibrillation. It is low cost (~30 €), wearable, slightly bigger (5 cm × 3 cm × 1.5 cm) than an everyday watch, wireless, and it does not require cables or disposable electrodes. Acquisitions are transmitted via Bluetooth to a custom smartphone app, where the recorded ECG is filtered to remove acquisition noise and baseline wandering. Then, the resulting signal is visualized and it can be shared via mail upon user request. Moreover, while parsing the ECG record, the embedded algorithm is able to automatically detect and signal atrial fibrillation episode. Finally, since it is a wearable device, it should also ensure, as much as possible, comfort when worn [18].

**Figure 1:**
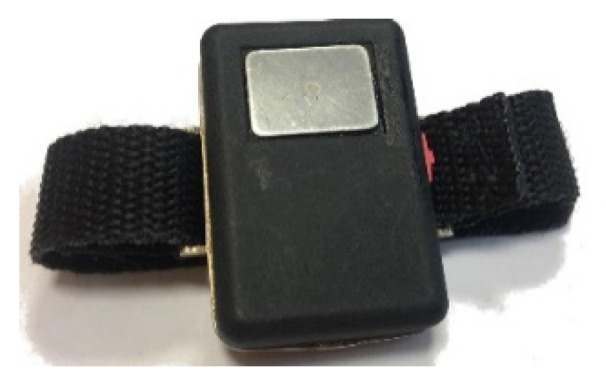
The ECG WATCH.

This paper presents an extension of [49], where the authors provided a general overview of the ECG WATCH and the initial experimental results. Here, the last version of the device, shown in Figure 1, is introduced and fully described with respect to its design specifications. Since it is a wearable device, its power consumption over time was fully assessed, and its accuracy was compared with the gold standard, both from visual and analytic perspectives. Finally, the algorithm for automatic atrial fibrillation detection was detailed and tested against a certified CVD simulator.

Section 2 presents state-of-the-art, commercially available, wearable devices for ECG recording. Section 3 describes the ECG WATCH, which is then compared with a certified electrocardiograph in Section 4. Finally, Section 5 yields the conclusions.

## 2. State of the Art

Portable devices for ECG acquisition are already available in the market [52], but only some of them can be used for both research and medical purposes with even less allowing the user to share results (e.g., via mail), and none of them provide the ECG trace in a numerical way. The majority of them are not wearable and their recording time is typically greater than 20 s, which makes them highly prone to muscular noise since acquisitions are not performed on the bed. An example is [53], which can acquire, one by one, the three peripheral leads (i.e., lead I, lead II, lead III), but it cannot print or share the recorded ECG. Furthermore, the quality of its recordings is not sufficient to be exploited from physicians to make a proper diagnosis.

A fast-growing class of devices is e-health, which are able to monitor and record several vital parameters of an individual, e.g., heart rate or blood oxygen level, using specialized hardware. E-health devices can be connected, e.g., via Bluetooth, to a smartphone, which can be exploited for user interface, data store, real-time analysis, network uplink, and its onboard sensors, such as accelerometers, cameras, and GPS [54].

The AliveCor [55,56], which is present both in the American and the European market (under different brands), can acquire a 30 s single-lead ECG and transmit it through an audio channel to the associated app. Then, the signal is processed to remove noise and search for any anomalies. It is a two-electrode device to be attached to the backside of the smartphone. Indeed, because of the chosen channel (i.e., audio transmission), it needs to operate very close to the phone. It is FDA approved and CE marked as a medical device. The output is a PDF file that can be shared by mail; as before, no numerical signal is accessible.

A similar device is [57], which uses Bluetooth protocol to transmit data instead of an audio protocol. It is FDA cleared and U.S. citizens can subscribe to a professional service that will check 30 s ECG recordings remotely. The output is a PDF file that can be shared by mail; no numerical signal is accessible.

Instead of a two electrodes bar to be used between two hands, QardioCore [58] proposes a chest belt to be worn under the clothes. The advantage of this approach is the possibility of a continuous ECG. It can also monitor a person’s physical activity and perspiration rate. It is FDA approved, CE marked, and clinically validated as a medical device. Although it is designed for endless acquisition, its design is better suited for usage during sport. Being worn constantly under clothes during everyday activities may result as uncomfortable. Finally, it works only with Apple iPhones.

The latter version of the Apple Watch [59] performs both heart rate computation and ECG recording using electrodes on its back and on the clock ring. Acquisitions can be stored into the watch or shared with Apple smartphones. It is wearable, wireless, and does not require any medical expertise to correctly acquire an ECG. Its 30 s ECG recording is FDA compliant. It only provides PDF files while ECG numerical trace is not accessible.

To conclude, to the best of our knowledge, all available solutions need a long recording time (not less than 20 s), which is adequate in stable situations, such as in an hospital bed, but more likely to be corrupted with noise during mundane activities. Furthermore, though it is undeniable—from a statistical point of view—that a paroxysmal atrial fibrillation with a few arrhythmias during the day can be better detected with long-time measurements, it is also true that a severe atrial fibrillation, typical of severe patients, is a continuous event. Thus, it is always detected in 10 s. Conversely, the ECG WATCH requires half of the acquisition time, in the worst-case scenario, with regards to other technologies. Moreover, as a side effect, shorter acquisitions also mean that it is less likely to corrupt the measurement with involuntary movements (i.e., muscle noise). Finally, unlike the available solutions, the ECG WATCH also provides results in a numerical format (besides a graphical PDF representation of the signal), which can be exploited for research activities and deeper medical analysis of the ECG traces (e.g., using different filtering or performing automated analysis with rhythm recognition algorithms).

## 3. The ECG WATCH

**Figure 2:**
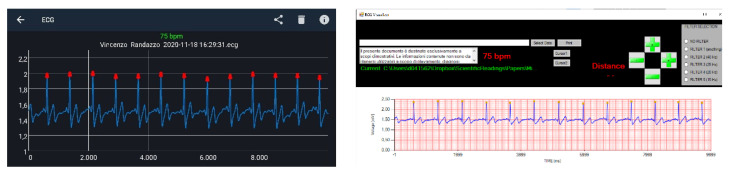
ECG visualization: mobile app (**left**); physician desktop software (**right**).

The ECG WATCH is a wearable, wireless, non-invasive device designed and built by the authors in the Neuronica Lab of the Politecnico di Torino. It is a heart monitor for easily recording 10 s single-lead ECG and visualizing it into a smartphone or desktop app. The recording can be sent to a physician who can analyze it and determine if the subject requires a deeper examination (see Figure 2).

The ECG WATCH is capable of acquiring skin biopotential through two electrodes: one placed on the front of the device, and one on the back. By touching the electrodes with different parts of the body (according to the Einthoven’s triangle [60]), it is possible to acquire one of the three ECG’s main derivations. Having a watch form factor means that, in normal operations, it is worn on the wrist. Therefore, the first electrode is in contact with one of the wrists, while the other one is free to be touched with the other hand, resulting in a lead I acquisition; or with the opposite leg, resulting in lead II or III acquisition (depending on whether the watch is worn on the left or right side).

The acquisition only lasts 10 s, and after that it is elaborated and transmitted via Bluetooth to the smartphone app. The application is the main way to interact with the acquired signals. Among various functions, it is mainly used for: filtering the signals coming from the device; storing the acquisitions in a database; displaying the signals; and sharing the acquisitions via e-mail. Moreover, the application has a built-in algorithm for atrial fibrillation recognition that monitors every acquisition sending an alarm in the case of an episode.

**Figure 3:**
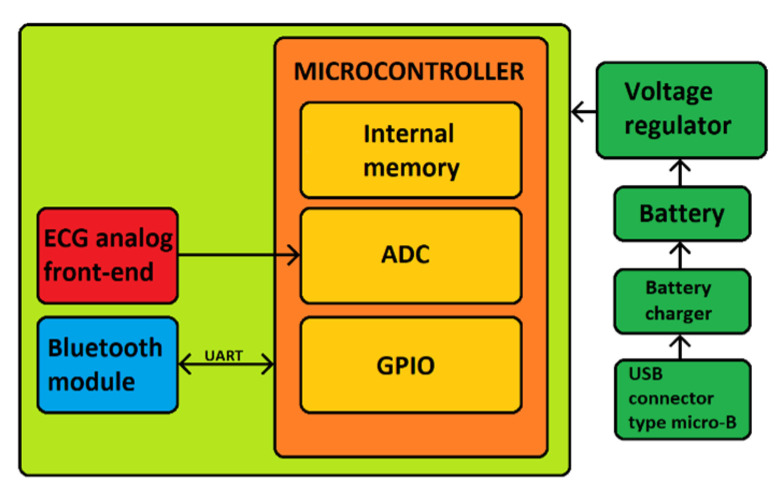
ECG WATCH block diagram: ADC—Analog-to-Digital Converter; GPIO—General Purpose I/O pins.

Figure 3 represents the block diagram of the ECG WATCH. A microcontroller powered by a LiPo battery is capable of acquiring an ECG signal through an analog front-end and transmitting the acquired data through a Bluetooth module. The next section will address the various elements in more detail.

### 3.1. Analog Circuit Design

**Figure 4:**
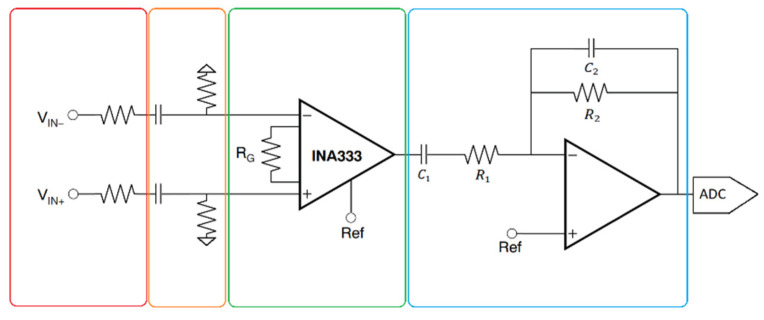
Analog chain. From left to right, the highlighted boxes contain: the electrodes, the passive high-pass filter, the differential amplifier, and the active band-pass filter.

The circuit used in the ECG WATCH (see Figure 4) takes inspiration by Thakhor and Webster [61] with the main difference of keeping particular attention on a lower power consumption and a smaller area. The good quality of the integrated circuit (IC) used in the front end, given by very high CMRR (Common Mode Rejection Ratio) and PSRR (Power Supply Rejection Ratio), makes the use of a right leg drive amplifier unnecessary [62]; albeit it could still be implemented for a less portable application because of the versatility of the circuit.

The analog front-end (see Figure 4) consists of a passive high-pass filter that feeds an instrumentation-amplifier (Texas Instruments, INA333), followed by an active band-pass filter. The acquisition chain ends with the microcontroller ADC. The instrumentation-amplifier is set with a gain of 40 dB, whilst the active band-pass has a 20 dB gain, for a grand total of 60 dB gain in the allowed band. Since most of the ECG spectrum is located beneath 70 Hz [63], the band-pass filter has been designed with a band of [0.7 Hz–72 Hz] with the following transfer function:

(1) $$H\left(s\right)=-\frac{R_{2}}{R_{1}}\frac{R_{1}C_{1}s}{R_{1}C_{1}s+1}\frac{1}{R_{2}C_{2}s+1}$$

which has a zero in the origin, two poles at −1/*R*_1_*C*_1_ and −1/*R*_2_*C*_2_, and a gain of −*R*_2_/*R*_1_. During the design phase, it was also taken into consideration to add a 50 Hz notch-filter in order to further eliminate the noise from the main-line coupling, but the results were already satisfying as they were.

The whole circuit is powered from a single 120 mAh LiPo battery that is regulated at 3.3 V by a buck/boost switching regulator operating at a relatively high frequency compared to the other signals. The 3.3 V is split in half (1.65 V) with a voltage divider coupled with a voltage follower OpAmp and used as a reference voltage. This reference is used as a virtual ground for the signal coming out of the INA333, so to avoid a double-ended power supply.

Finally, the front-end is connected to the patient through two small (2 × 2 × 0.1 cm) stainless steel electrodes. This material offers the best compromise in terms of costs, usability, signal stability, and mechanical resistance.

**Figure 5:**
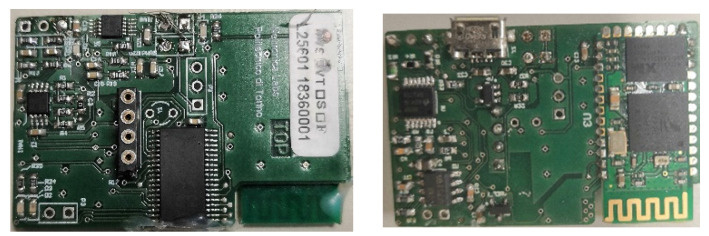
The mounted PCB: top (**left**) and bottom (**right**).

**Figure 6:**
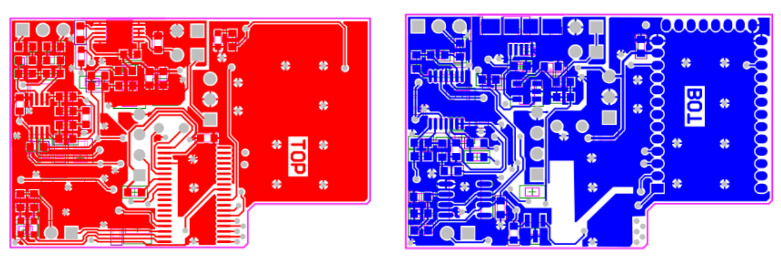
The PCB Gerber view: top (**left**) and bottom (**right**).

In Figure 5 and Figure 6, the analog front-end is all concentrated in the extreme left of the board, occupying just 90 mm^2^ of board space (not including the electrodes connector).

### 3.2. Digital Circuit Design

The ECG signal is sampled at 1 kbps by the TI MSP430 microcontroller (µC) which has a 10 b 200 kbps SAR ADC on board, whose voltage reference is provided by an external component.

Ten seconds of recording are sufficient to the app embedded algorithm for assessing the risk of an atrial fibrillation; however, the µC flash memory is big enough to memorize on board a grand total of 70 s of ECG sampled at 1 kbps, removing the need of an additional memory unit and, therefore, saving some space on the board.

Since the application is not time critical, to further reduce the printed circuit board (PCB) dimensions, the µC works at 16 MHz using its internal oscillator. The µC computational power is far beyond the actual needs of the application. As a consequence, some digital signal processing could be performed directly on board [64] and it is actually under study. Figure 5 and Figure 6 yield the PCB: on the bottom (see Figure 5 and Figure 6 right), there are the connectors for the battery, the two electrodes, and the USB recharger; while the top, shown in Figure 5 and Figure 6 left, houses the µC and other components of the front end.

### 3.3. Power Consumption

Most of the power consumption depends on the digital and power circuits, which grossly absorbs 30 mW for the recording and 150 mW for the brief Bluetooth data transmission. The analog circuit only draws approximately 1.5 mW due to the extremely low-power OpAmp and InAmp employed in the design. In fact, the chosen ICs (Texas Instruments OPA4330 and INA333) combine very low power consumption with very high performances in terms of PSRR, CMRR (for the INA333), offset, drift, noise, and an internal EMI filter. Using a standard 120 mAh single-cell LiPo battery ECG WATCH has an estimated battery life of 8 days, assuming a heavy use of 50 acquisitions per day.

Finally, the user can recharge the device with a standard USB type micro-B cable, vastly employed for charging smartphones and, therefore, widely available.

## 4. Experiments

To assess the quality of ECG WATCH acquisitions, a comparative study with a standard three-lead patient monitor was conducted on 30 people (15 males, 15 females) aged 25–35 years old with no cardiac problems, and with a patient simulator (Fluke Biomedical ProSim 4). The apparatus chosen for the comparison is the GE Healthcare patient monitor B105, a CE medical device employed by doctors in hospitals or infirmaries.

Three channels, four electrodes, ECG recordings were taken using pre-gelled Silver-Silver Chloride (Ag/AgCl) electrodes as standard for ECGs comparison in the PolitoBIO Med laboratory of Politecnico di Torino. ECG WATCH acquisitions were taken among wrists, except in five cases (two males, three females), where lead I’s signal was too weak (not clearly visible) and acquisitions were taken between the right arm and the left leg, i.e., lead II. The choice of acquiring lead II’s signal was decided by the research team. The associated procedure will be detailed in the instruction manual. As post-processing three different filters were used: a baseline-wander removal filter, a notch filter used to remove 50 Hz noise, and low-pass moving average filter to smooth the results. The acquisitions were taken simultaneously. Then, the ECG WATCH and B105 recordings were manually aligned using time as a reference.

**Figure 7:**
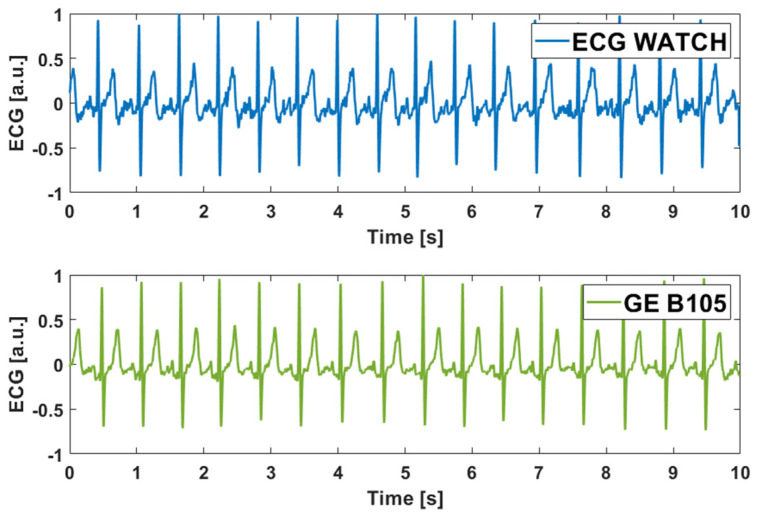
Comparison between ECG WATCH (**top**) and GE Healthcare B105 (**bottom**) on a single subject lead I.

Figure 7 compares the recordings on a single subject of the ECG WATCH (blue) and the B105 (green). Qualitatively speaking, it can be stated that the ECG WATCH acquisition is quite similar to the gold standard one. This is, of course, just a qualitative example of the accuracy of the ECG watch compared to B105, and the rest of this section will focus more on the quantitative aspect. In this sense, the ECG WATCH aims to be considered as a medical device and, in fact, the CE medical certification process has already begun.

### 4.1. Bland-Altman Plot

**Figure 8:**
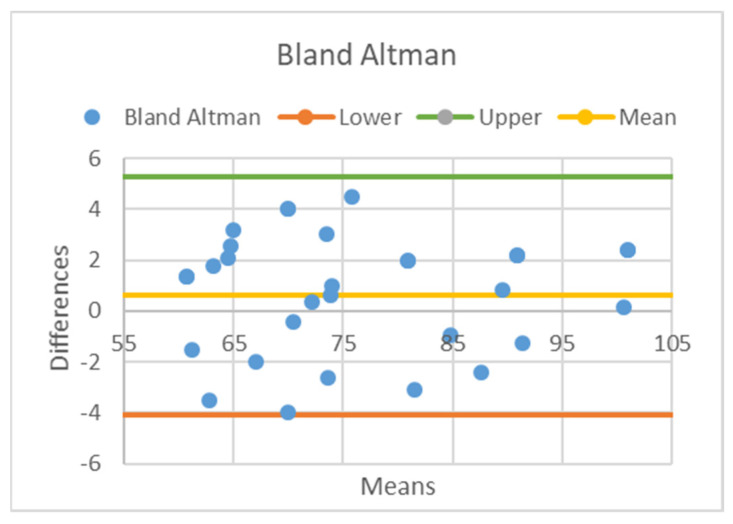
Bland-Altman plot for the GE Healthcare B105 and the ECG WATCH: each blue point represents a pair of heart rate calculations; yellow line represents the average difference, while orange and green lines represent the lower and upper bounds of the 5% fiducial interval.

The heart rate computation has been the first criterion for evaluating the ECG WATCH quality with respect to the GE Healthcare B105. The difference between the two devices was determined with the Bland-Altman plot (BA plot) [65] shown in Figure 8: each blue point represents a pair of heart rate calculations and provides an estimation of the discrepancy between the two devices for a given simultaneous acquisition; differences between couples of measurements are plotted in ordinates, while their corresponding means are drawn in the abscissa. The BA plot avoids uncertainties of different measurements because the estimation is evaluated on the differences of a single couple of acquisitions; thus, recording conditions (e.g., heart rate variability) do not interfere with the results. In this sense, the BA plot is a descriptive statistical tool for comparing two devices.

From Figure 8, it clearly stems out measurements are biased of only 0.6 heartbeat (HB) per minute, which means ECG WATCH overestimates, on average, the HB by 0.6 bpm. However, data are consistent because they vary in an interval of less than 5% of the mean value. Furthermore, the cross correlation between the two heart rate estimations is around 98.7% with an average standard deviation for each patient of 2 bpm. As a consequence, the ECG WATCH can be considered as a valid tool to compute heart rate, which is one of the key parameters monitored by cardiologists to assess the cardiac state of health.

### 4.2. Power Spectral Density (PSD)

The ECG WATCH is not only a heart rate monitor but mainly an ECG recorder; therefore, the second benchmark is the ECG quality. As explained in [49], despite the amount of instrumentation and knowledge on ECG, asserting its quality is not trivial, especially from an analytical perspective. Here, the ECG WATCH acquired signal was evaluated both in the frequency and time domains.

The power spectral density (PSD) provides information on the signal power distribution among the spectrum; in this sense, it can be considered as the information content of each frequency. For the sake of simplicity, among the different techniques for estimating a signal’s PSD, the squared discrete fast Fourier transform (FFT) module has been employed:

(2) $$PSD\left(f\right)=\frac{{\left(\Delta t\right)}^{2}}{T}{\left|\overset{N}{\underset{n=1}{\sum }}x_{n}e^{-i\omega n\Delta t}\right|}^{2}$$

**Figure 9:**
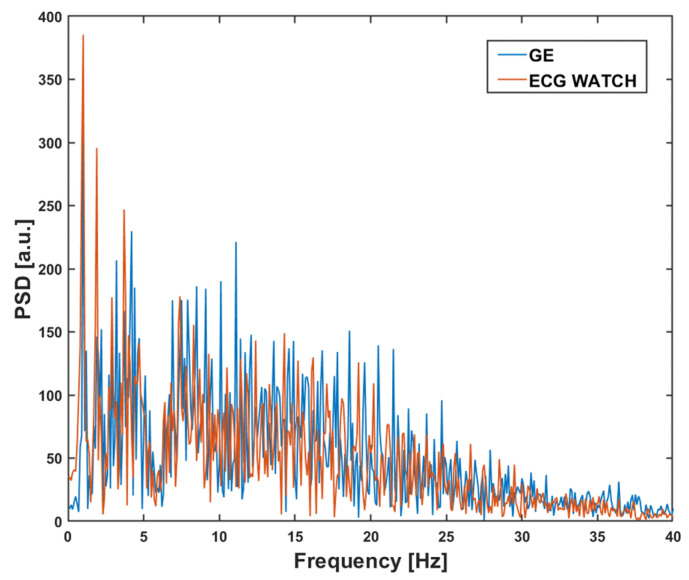
Power spectral density comparison in the band 0–40 Hz for the GE Healthcare B105 (blue) and the ECG WATCH (red).

Figure 9 yields the comparison between the PSDs for the ECG WATCH and the B105.

Despite there being no visual significant difference between the two densities, an additional analytical study was performed using cumulative spectral power (CSP), which is derived from PSD as a cumulative sum normalized with the total power. The resulting curve, called *CSP*(*f*), is a monotone function to represent the percentage of energy encompassed in the spectrum within a specific frequency *f*:

(3) $$CSP\left(f\right)=\overset{f}{\underset{n=1}{\sum }}PSD\left(n\right)$$

The argument *f* can be exploited to derive at which frequency the signal reaches a certain amount of the total power and thus of the information content. In this sense, it can be defined as the median, i.e., the frequency, that splits the power in half, and a bandwidth around it, which has been set to 60%. The CSP formula has been applied to 90 acquisitions taken from 30 anonymous volunteers (3 per patient), and 30 acquisitions taken using ProSim4 simulating both normal sinus rhythm and atrial fibrillation. All acquisitions have been performed at the same time for both ECG WATCH and B105 to have consistent results. In order to obtain an idea of the short-term differences between the devices, Table 1 contains the comparative results for some of the acquisitions.

However, to have a better understanding of the system performance in terms of multiple acquisitions, Table 2 shows the average values of the frequencies at which 20%, 50%, and 80% of total power is distributed, according to CSP.

The values in Table 2 validate the information content of the ECG WATCH and B105 is distributed in a similar way, in according with Figure 9: the ECG WATCH has a spectrum concentrated on slightly lower frequencies than the B105, where the great part of the ECG information is located [66].

### 4.3. Signal to Noise Ratio (SNR)

Another means of evaluation based on the frequency domain is the signal to noise ratio (SNR). It is defined as the ratio of signal power to the noise power, and it is usually expressed in decibel (dB):

(4) $$SNR=\frac{P_{signal}}{P_{noise}}$$

By definition, signal and noise are the meaningful and the unmeaningful information, respectively, and are chosen arbitrarily depending on the system to evaluate.

For this comparison, it has been defined as signal, i.e., meaningful information, everything in the bandwidth of 0.67–40 Hz as stated in IEC 60601-2-27 regarding electrocardiographic monitoring instruments, and noise as everything lying outside that frequency band. Thus, *P_signal_* and *P_noise_*, in Equation (4), are referred to the sum of the power spectral density (Equation (2)) evaluated inside and outside the significative bandwidth [0.67–40] Hz, respectively.

The results are summarized as mean and standard deviation in Table 3. The same considerations regarding the data in Table 2 also apply for Table 3.

Albeit, the results show that the ECG WATCH has a slightly lower SNR than the B105, it has less variability, which means the information content of its acquisitions is more consistent in the considered bandwidth. Moreover, a difference of 17 dB on average is not very significant when the values are way above 100 dB.

### 4.4. Time-Domain Differences

**Figure 10:**
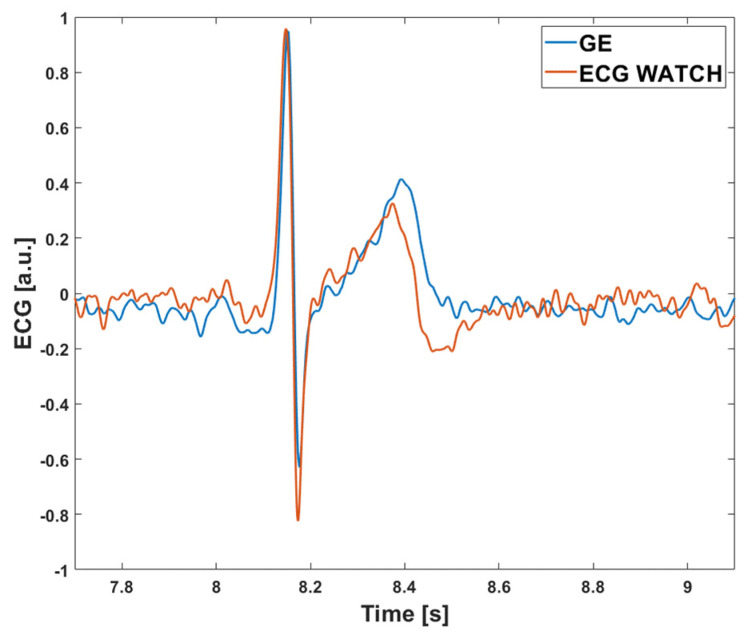
Single heartbeat isolated from ECG WATCH (red) and GE Healthcare B105 (blue) contemporary acquisition.

The final comparison between the ECG WATCH and B105 was performed in the time domain. To this purpose, a dataset made of single HBs randomly extracted from volunteers was used for evaluating point-to-point discrepancies of the two measurement devices. The contemporary acquired signals from the B105 and ECG WATCH were first normalized, then matching HBs were isolated and compared in pairs. To better explain the concept, Figure 10 shows an example of such couple of heartbeats: one acquired with the ECG WATCH (in red), and the other one with the B105 (in blue).

Table 4 reports the average, the standard deviation, and the maximum value of the difference between each point of the signals normalized to 1: it can be observed that there are not significative differences, with a mean value below 3%, and a standard deviation around 9%.

## 5. Atrial Fibrillation Detection

One of the most frequent, dangerous, and hard to detect cardiac pathologies is atrial fibrillation. According to [67], A-fib is an abnormal heart rhythm where heart atrial chambers beat with a rapid and irregular pace. It can remain silent, i.e., without any symptoms [68], for years and undetected even by professional tools. Indeed, it often begins as a few abnormal beatings which become more frequent over time [69]. Occasionally there may be symptoms, such as heart palpitations, fainting, lightheadedness, shortness of breath, or chest pain [70]. Furthermore, a heart beating in such an irregular way increases the risk of heart failure, dementia, and stroke [67].

**Figure 11:**
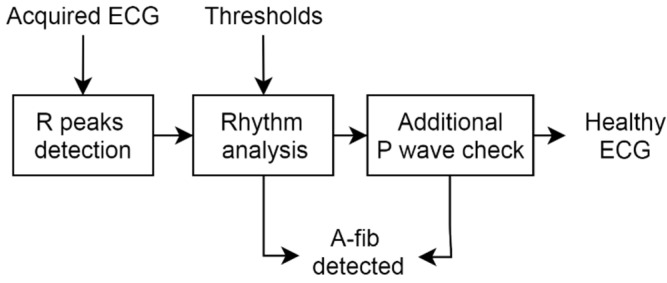
A-fib detection algorithm: block diagram.

The ECG WATCH is sized as small as a watch, to be worn on wrist, and needs just a tap on a phone app to record a 10 s ECG, that is, to check cardiac health. It does not require any particular expertise, e.g., medical, to be used; therefore, the ECG WATCH is perfectly suitable to perform a heart check anytime, anywhere. For this purpose, the app embeds an algorithm for automatically detecting an atrial fibrillation (see Figure 11). At first, the R peaks, i.e., the heartbeats, are extracted from the 10 s recorded ECG using the well-known Pan–Tompkins algorithm [71]. Then, both the beat-by-beat and overall rhythm are analyzed to check if their variations over time exceed the predefined thresholds (experiments showed that a good value is around 3 bpm); if so, the recording is classified as A-fib. On the contrary, if the rhythm is considered as “normal”, a final check on the P wave is performed. As is well known in medicine, in the case of atrial fibrillation, P waves will be absent. However, some people with A-fib will have fibrillatory waves, i.e., a wavy baseline, on their ECG, which signal atria pulse irregularly. They may resemble P waves, and this can make an A-fib rhythm look like a sinus one. The final block of the algorithm looks for P waves by means of the highest maxima before the R-peak. When it found a wave resembling a P wave, its amplitude, duration, and distance from previous and subsequent QRS complexes are checked to determine if it is a true P wave or a fibrillatory one.

### Algorithm Assessment

**Figure 12:**
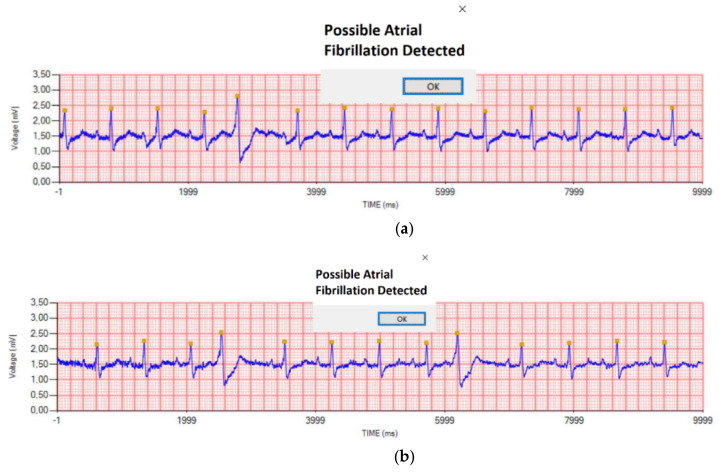

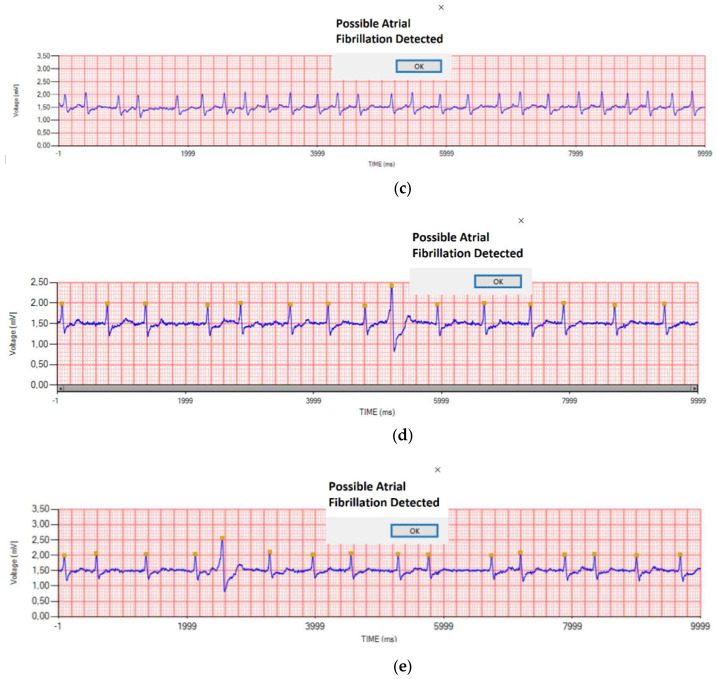
A-fib detection algorithm: real subject examples. The ECG WATCH desktop software analyzes the recording and automatically detects and signals the A-fib (see popup messages).

The A-fib algorithm has been tested both on real and simulated recordings. Figure 12 shows some examples of 10 s arrhythmic ECGs taken from real subjects: the disease is always correctly recognized and signaled (see the pop-up messages) by the desktop software. In this sense, it can be stated that in the case of severe A-fib, ten seconds are sufficient for detecting this pathology and the ECG WATCH proves to be a valid tool for heart monitoring.

**Figure 13:**
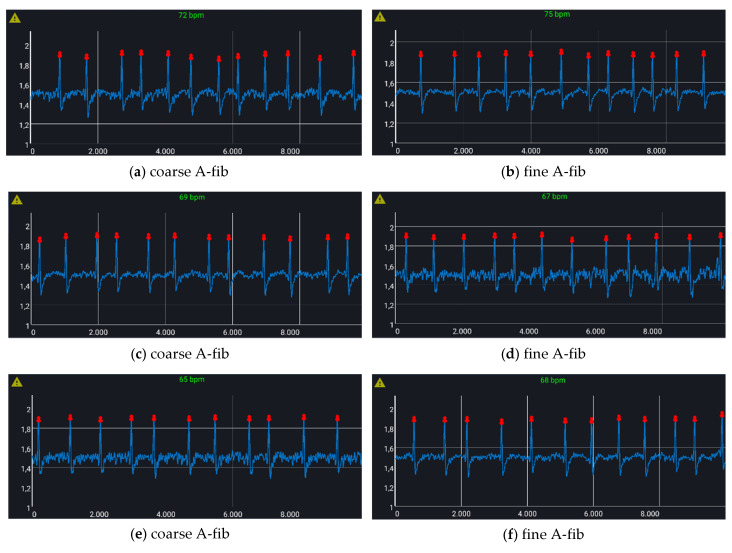
Fluke Biomedical ProSim 4 simulated ECGs: coarse (**left**) and fine (**right**) A-fibs. The ECG WATCH mobile app signals the detected anomaly by means of a yellow triangle on the top left corner and of an acoustic alarm signal.

In order to assess the algorithm quality, a stress test was performed with the use of a certified standard simulator, the Fluke Biomedical ProSim 4, which is able to produce, among the others, both healthy and atrial fibrillation ECG signals. Figure 13 shows some examples: either coarse (Figure 13a,c,e) or fine (Figure 13b,d,f) A-fibs were tested. The algorithm was able to correctly identify all the pathological traces as dangerous ones; thus, the mobile app generates an alert for the users by means of a yellow triangle on the top left corner and of an acoustic alarm signal.

**Figure 14:**
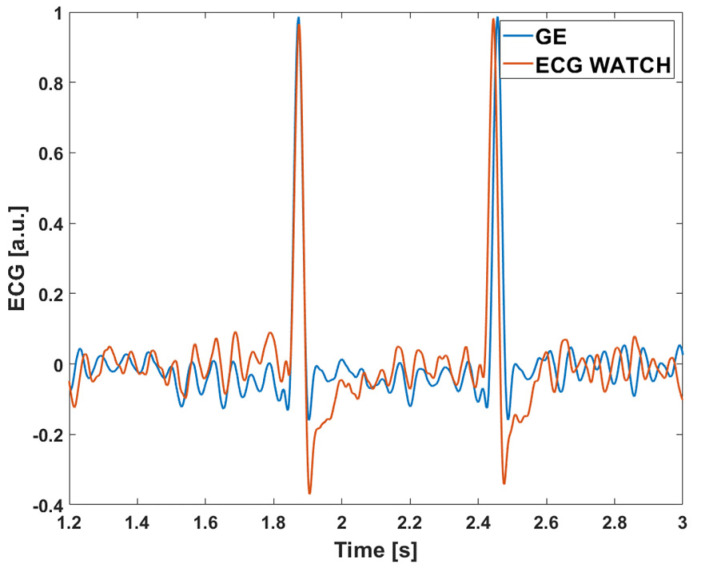
Fluke Biomedical ProSim 4 simulated ECGs. Comparison between GE Healthcare B105 (blue), and ECG WATCH (red) recordings.

Finally, Figure 14 compares the ECG WATCH (in red) and the GE Healthcare B105 (in blue) acquisitions on a simulated atrial fibrillation signal. As the previous case, ECG WATCH recording is compatible with the GE Healthcare B105 one.

## 6. Conclusions

Cardiovascular diseases characterized by occasional ECG anomalies, like atrial fibrillation, are difficult to be detected. Current solutions such as Holter or wearable devices fail to properly tackle these pathologies. Indeed, despite they can detect some episodes, they are either not wearable or wireless and are often unsuitable for a long-time usage. In addition, the quality of recordings is another key factor to perform reliable CVD diagnosis. The ECG WATCH is designed to solve all the above-mentioned problems at the same time; it is a low-cost, wearable, wireless, unobtrusive device for acquiring ECG in only 10 s, anytime, anywhere. It can acquire any of the peripheral leads and send the recordings to doctors by just tapping a button on a smartphone app. It does not require any medical expertise to be positioned or used. The quality of proposed tool has been successfully assessed on 30 people with respect to a certified electrocardiograph. Furthermore, the ECG WATCH requires at least half the acquisition time of other commercially available tools, and its numerical output can then be exploited by a cardiologist for deeper inspection and analysis, as it was shown in the experimental section. The app embeds an algorithm for A-fib detection, which was successfully tested with a certified ECG simulator on different severities of the pathology. Finally, the proposed device is also low-cost, which allows its adoption on a very large population.

In conclusion, the ECG WATCH has proved to be an interesting and promising device for anytime cardiac health monitoring and for detecting silent atrial fibrillation without the need for medical expertise or going to a doctor. Future works will deal with device size reduction and an extension of the embedded algorithm for detecting more pathologies.

## 7. Patents

The device presented here is based on patent WO2018073847A1: wearable device for acquiring electrocardiographic signals (ECG) signals.

## Figures and Tables

**Table 1:** Comparison between ECG WATCH and B105 cumulative Spectrum Power frequencies for paired acquisitions.

ECG WATCH f 20% [Hz]	B105 f 20% [Hz]	ECG WATCH f 50% [Hz]	B105 f 50% [Hz]	ECG WATCH f 80% [Hz]	B105 f 80% [Hz]
3.4	3.4	9.8	9.8	17.0	17.1
1.7	2.5	7.6	7.8	15.7	15.9
3.2	3.6	8.2	7.7	14.6	13.9
4.6	4.2	10.1	9.7	17.5	17.7
4.5	4.2	8.8	7.5	15.5	15.2
1.6	1.2	4.0	3.7	12.9	12.4
4.3	3.8	9.2	9.0	16.6	17.1
3.2	3.2	9.7	9.8	16.8	16.7
3.6	3.6	10.5	8.6	16.1	14.6
3.8	1.9	9.1	7.3	16.4	15.2

**Table 2:** Cumulative spectrum power frequencies.

System	f 20% [Hz]	f 50% [Hz]	f 80% [Hz]
B105	3.9	8.7	15.3
ECG WATCH	3.6	8.6	15.3

**Table 3:** Signal to noise ratio (SNR).

	Mean [dB]	Standard Deviation [dB]
B105	145.7	27
ECG WATCH	128.14	10

**Table 4:** Time domain differences.

	Mean	Standard Deviation	Max
Differences	−0.027	0.0931	0.1508

## Data Availability

The data presented in this study are available on request from the corresponding author.
